# Effects of Increased Housing Space Without Altering Stocking Density on Body Weight, Stress, and Gut Microbiome in Broiler Chickens

**DOI:** 10.3390/ani15030441

**Published:** 2025-02-05

**Authors:** Eun Bae Kim, Seojin Choi, Jongbin Park, Biao Xuan

**Affiliations:** 1 Department of Applied Animal Science, College of Animal Life Sciences, Kangwon National University, Chuncheon 24341, Republic of Korea; chlrlqoqkqh@naver.com (S.C.); jong7bin@gmail.com (J.P.); xuanbiao@ybu.edu.cn (B.X.); 2 Institute of Animal Life Science, Kangwon National University, Chuncheon 24341, Republic of Korea; 3 Microbiome Convergence Research Center, Korea Research Institute of Bioscience and Biotechnology (KRIBB), Daejeon 34141, Republic of Korea; 4 Department of Animal Science, College of Agriculture, Yanbian University, Yanji 133002, China

**Keywords:** broiler, stocking, gut microbiome, stress, chicken, body weight, *FKBP51*

## Abstract

Effective poultry management practices that promote chicken health are crucial for producing higher-quality chicken meat at a lower cost. This study examined the hypothesis that increasing space while maintaining stocking density may positively impact poultry health. We evaluated body weight (BW) as an indicator of growth, stress markers, and the composition of the gut microbiome by comparing two housing sizes: smaller (control) and larger (treatment) spaces, with 10 birds per space and a stocking density of 12.3 birds/m^2^. Chickens in the larger space had 15% higher BW (*p* = 0.06) compared to those in the smaller space when significance was evaluated at *p* < 0.10. Stress indicators such as blood cortisol (acute) and brain *FKBP51* did not differ significantly. Faith’s phylogenetic diversity was marginally higher in the larger space (*p* = 0.05), and microbial communities differed significantly between the two groups. The relative abundance of several genera, including *Clostridium_sensu_stricto_1* (*p* = 0.02), *Lactobacillus* (*p* = 0.03), and *Paracoccus* (*p* < 0.01), was greater in the larger space, whereas *Turicibacter* (*p* = 0.02), *Escherichia–Shigella* (*p* = 0.01), and *Lysinibacillus* (*p* = 0.01) were more abundant in the smaller space. The larger and smaller spaces were associated with a significant (*p* < 0.05) increase in the abundance of 39 and 25 MetaCyc pathways, respectively, involved in amino acid and nitrogen metabolisms. These findings suggest that increasing housing space without altering stocking density or additional treatments may improve both growth and gut microbiome health in broilers. Our results provide insights into the relationship between chicken housing environments and the gut microbiome.

## Simple Summary

This study looked at whether giving chickens more space to live could improve their health and growth without additional treatments. Two different housing sizes were compared for chickens: larger and smaller spaces. Chickens in the larger space were heavier, although stress levels were similar in both groups. The chickens in the larger space also had healthier gut bacteria, which might be important for digestion and overall health. Certain beneficial bacteria were more common in the larger space, which might help with chicken metabolism. This study suggests that providing more space for chickens, without increasing the number of birds, can lead to better growth and a healthier gut.

## 1. Introduction

In the poultry industry, broiler production is essential for supplying a significant amount of protein for human consumption [1]. As broiler production has increased, considerable efforts have been made to enhance the breeding conditions of chickens to promote animal welfare. In particular, researchers and farmers have been striving to produce healthier broilers by improving stocking density while maintaining economic viability [2].

High stocking density (HSD), which is commonly used in intensive livestock farming, has been associated with several practical challenges. For instance, previous studies have shown that HSD reduces feed intake and BW gain [3,4], while also decreasing alpha diversity and the abundance of commensal or beneficial bacteria in the gut microbiome [5] due to increased stress. To mitigate these adverse effects, two primary strategies have been employed: supplementing feed additives or reducing stocking density, both of which aim to alleviate density-induced stress. The administration of feed additives under HSD has been shown to enhance both productivity and health in chickens [3,5,6,7]. For example, chlorogenic acid has been reported to improve gut integrity, gut microbiome, and oxidative stress in broilers raised under HSD [5,6]. Reducing stocking density, however, requires additional costs for facilities or land to accommodate a larger housing space. Moreover, even at a reduced stocking density, broilers may still experience relative discomfort, as indicated by their preference for the wall areas [8].

The gut microbiome is a diverse community of microorganisms living in the gastrointestinal tract (GIT) [9,10,11]. This microbial community includes both commensal and pathogenic microorganisms. Its composition is significantly influenced by various factors such as diet, nutrition, stress, immune responses, and habitat conditions like housing density or size. Some gut microorganisms are known to be linked to the health and diseases of animal hosts, including chickens and humans [1,3]. The gut microbiome can even influence mental health through the microbiota–gut–brain axis [12], though this connection is not yet fully understood [2,3]. Based on this concept, alterations in the gut microbiome of chickens under the specific housing environments described above may serve as important biological indicators of mental health.

As previously described, reducing stocking density still results in relative discomfort [8], which may influence growth and the gut microbiome in broilers. Expanding the housing area while maintaining a lower stocking density may provide broilers with a more spacious central area in the pen for resting and movement [8], potentially reducing competition and stress. Based on this premise, we hypothesized that such a simple environmental intervention, without additional treatments, could enhance growth, alleviate stress, and positively influence the gut microbiome in broilers. To test this hypothesis, we conducted a small-scale pilot trial to investigate the impact of housing space expansion on growth, stress, and the gut microbiome dynamics.

## 2. Materials and Methods

### 2.1. Animal Trials

We obtained 200 one-day-old broiler chicks (Ross 308, day 1) from a local poultry hatchery and transported them to the animal experimental farm at Kangwon National University (Chuncheon, Republic of Korea). After one day of acclimation in the facility, we selected twenty chicks based on their initial BW (42.9 ± 0.7 g). They were divided into two groups: one housed in a larger pen (*n* = 10; dimensions: 1400 mm × 580 mm) and the other in two smaller pens (*n* = 5 per pen; dimensions: 700 mm × 580 mm each). All groups were kept under identical conditions, including a flat floor and rice hull bedding, with the same stocking density of 12.3 birds/m^2^ for each pen. Temperature and relative humidity were continuously monitored using sensors (DHT22, Guangzhou AoSong Electronics Co., Ltd., Guangzhou, China) connected to a microcontroller (NodeMCU, Ai-Thinker (Shenzhen, China)), with averages of 27.4 ± 2.0 °C and 68.8 ± 9.9%, respectively, and ranges from 21.9 to 33.6 °C for temperature and from 42.5 to 94.6% for humidity. The chicks had ad libitum access to fresh water and a commercial starter feed until they were 14 days old (day 14), followed by a commercial well-textured pellet feed until they reached 39 days of age (day 39). The nutritional information for the two feeds is the same as previously reported [13]. This animal experiment was conducted in accordance with the guidelines approved by the Institutional Animal Care and Use Committee (IACUC) of Kangwon National University (KW-210611-1).

### 2.2. Sample Collection

On day 39 of the experiment, the BWs of the broilers were measured, and fecal samples were collected. Blood samples were also collected from the wing veins using a 1-mL sterile syringe without anticoagulant. The samples were then stored in 1.7-mL polypropylene microtubes at 4 °C for approximately 30 min before being centrifuged at 3000× *g* for 5 min to obtain serum. The broilers were then euthanized using CO_2_ in a chamber, and the frontal parts of their brains were isolated. All collected samples, including feces, serum, and brains, were immediately frozen in liquid nitrogen after collection and transported to the laboratory, where they were stored at −80 °C in a deep freezer until further analyses.

### 2.3. Extraction and Sequencing of Fecal Microbial DNA

Fecal microbial DNA was extracted from 250 mg of feces, including the gut microbiome, by using the NucleoSpin Soil kit (Macherey–Nagel, Düren, Germany) according to the manufacturer’s instructions. The isolated DNA was stored at −20 °C until further analyses. The V4 region of the 16S rRNA gene was amplified by PCR with a set of specific primers (forward: 5′-GGACTACHVGGGTWTCTAAT-3′; reverse: 5′-GTGCCAGCMGCCGCGGTAA-3′) and Ex-Taq DNA polymerase (Takara Bio, Shiga, Japan). The amplification conditions were 94 °C for 3 min, followed by 30 cycles of 94 °C for 45 s, 55 °C for 1 min, and 72 °C for 1.5 min, with a final step at 72 °C for 10 min [14]. The PCR amplicons were purified, pooled, and sent to a sequencing company (eGenome, Seoul, South Korea) for library preparation and sequencing on the Illumina MiSeq by generating paired-end reads of 2 × 300 bp.

### 2.4. Microbial Community Analysis

To analyze the microbial community in broiler gut samples, we employed Quantitative Insights into Microbial Ecology 2 (QIIME 2, version 2024.2, https://qiime2.org, accessed on 3 March 2024) software [15] as described previously [13]. Raw Illumina-sequenced reads were processed for primer and adapter removal, as well as demultiplexing, by using custom Perl scripts and QIIME2 plugins such as cutadapt and demux. By using the DADA2 plugin, the reads were subsequently quality trimmed, filtered, and chimeric sequences were removed. Based on an rRNA sequence database, SILVA [16], operational taxonomic units (OTUs) were picked, and taxonomic assignments were conducted for each representative read. Alpha diversity indices and beta diversity were calculated based on a phylogenetic tree by using QIIME 2 core-metrics-phylogenetic, alpha-group-significance, and beta-group-significance options. Alpha diversity indices such as observed features and Faith’s phylogenetic diversity (Faith’s PD) was calculated with 8000 effective reads per sample to assess species richness and phylogenetic diversity, respectively. Beta diversity, indicating community dissimilarity, was determined using weighted or unweighted UniFrac distances based on all effective reads per sample.

### 2.5. Prediction of Metagenomic Pathways

Based on 16S rRNA reads, functional pathway predictions were generated using QIIME2’s q2-picrust2 plugin (https://github.com/gavinmdouglas/q2-picrust2, accessed on 15 July 2024), which is for the Phylogenetic Investigation of Communities by Reconstruction of Unobserved States (PICRUSt) [17]. In this analysis, we focused on the results for the MetaCyc metabolic pathways database [18].

### 2.6. Analysis of Blood Cortisol

Broiler serum samples were sent to Seegene Medical Foundation in Seoul, South Korea for blood cortisol analysis. Blood cortisol levels were measured by an electrochemiluminescence immunoassay (ECLIA) performed with the Cobas e 801 module (Roche Diagnostics, Mannheim, Germany) and Elecsys Cortisol II (Roche Diagnostics, Mannheim, Germany).

### 2.7. RNA Extraction of the Brain

To extract RNA, the frontal part of each brain tissue (~500 mg) stored in a deep freezer was placed in a bowl with liquid nitrogen and ground into a powder. The powdered tissue samples (~70 mg each) were collected, and 750 µL of TRIzol reagent (Thermo Fisher Scientific Inc., Waltham, MA, USA) was added, followed by a 10-min incubation. Next, 150 µL of chloroform was added, the mixture was vortexed for 15 s, and then incubated at room temperature for 10 min. After centrifugation at 3000× *g* for 15 min at 4 °C, the supernatant was transferred into a new tube. Subsequently, 500 µL of isopropanol was added, and the mixture was incubated overnight at −20 °C. The mixture was then centrifuged at 3000× *g* for 30 min at 4 °C, and the supernatant was discarded. The resulting white pellet was washed with 1 mL of 70% ethanol, centrifuged again at 4 °C for 30 min, and the supernatant was removed. The pellet was air-dried for 10 min at room temperature, dissolved in DEPC-treated water, and incubated at 50 °C for 5 min.

### 2.8. cDNA Synthesis and Quantitative Real-Time PCR

The extracted RNA was used to synthesize cDNA using the PrimeScript™ RT Reagent Kit (Perfect Real Time) (Takara Bio, Shiga, Japan). Quantitative real-time PCR (qPCR) was conducted with TB Green Premix Ex Taq II (Tli RNaseH Plus) (Takara Bio, Shiga, Japan) to target a housekeeping gene (glyceraldehyde 3-phosphate dehydrogenase, *GAPDH*) and a stress-related gene (FK506-binding protein 51, *FKBP51*, also known as *FKBP5*). Two primers for *GAPDH* were 5′-GTGGTGCTAAGCGTGTTATCATC-3′ (forward) and 5′-GGCAGCACCTCTGCCATC-3′ (reverse) [19], designed from NCBI Accession No. NM_204305.2 and used to produce a 269 bp amplicon. The other two primers for *FKBP51* were 5′-GCGAGGACCTGTTTGAGGAT-3′ (forward) and 5′-TGCAGTCAAAGCGAGTTCCA-3′ (reverse) from NCBI Accession No. AY723747.1 and were used to produce a 135 bp amplicon. The mRNA expression levels were quantified using the delta-delta Ct method, with *GAPDH* Ct values as the internal controls and *FKBP51* Ct values as the target.

### 2.9. Statistical Analysis

Statistical analyses were performed using R (version 4.3.1 or later). To compare mean values between two groups, either Student’s *t*-test or the Mann–Whitney U test was applied after assessing the normality of each group using the Shapiro–Wilk test. Statistical significance was determined at *p* < 0.05 (in most cases) or *p* < 0.10 (in some cases). Pearson correlations were calculated, with significance evaluated at *p* < 0.05.

## 3. Results

### 3.1. Physiological Changes in Broilers

**Figure 1:**
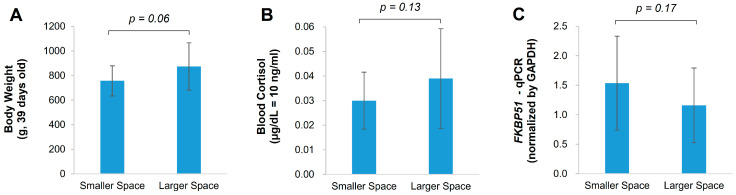
Physiological parameters of 39-day-old chickens. Body weight (**A**) was measured to assess chicken growth. Blood cortisol (**B**, µg/dL) and *FKBP51* mRNA expression (**C**) were measured to evaluate stress levels. Bars represent mean values with standard deviations indicated by error bars. The *p*-values were calculated using a one-tailed Student’s *t*-test, following an assessment of normality with the Shapiro–Wilk test.

The effects of housing size were evaluated by comparing physiological parameters such as BW, blood cortisol, and brain *FKBP51* mRNA expression. Interestingly, broilers housed in the larger space had a higher body weight (BW) (874 ± 192 g) compared to those housed in the smaller space (757 ± 122 g) (*p* = 0.06, Figure 1A). This difference of 117 g represents a 15.4% increase in average BW relative to broilers in the smaller space, which is not negligible from an economic perspective. Therefore, we considered this difference statistically significant at the *p* < 0.10 level. Contrary to our initial expectation that the smaller space would increase stress levels in broilers, the blood cortisol levels, which can acutely increase during sampling, did not show a significant difference (*p* = 0.13, Figure 1B). The cortisol levels were 0.03 ± 0.01 µg/dL in the smaller space and 0.04 ± 0.02 µg/dL in the larger space. The expression level of the *FKBP51* gene did not exhibit significant regulation in response to housing size (*p* = 0.17, Figure 1C). These findings suggest that while housing size influenced the BW of broilers, even at the same stocking density, it did not have a significant impact on their stress levels, which were likely acute.

### 3.2. Sequence Statistics of Gut Microbiome

The broilers’ gut microbiome on the final day was analyzed by sequencing 16S rRNA genes from fecal samples. Due to sample loss during storage, only eight samples from the smaller space and ten samples from the larger space were used. A total of 637,994 reads (*n* = 18) were obtained from the sequencing. After quality filtering, denoising, and chimera checking, 546,949 reads (85.7% of the initial reads) remained. The average reads per sample were 31,807 ± 13,684 (*n* = 8; 20,236 to 57,959 reads) for the smaller space and 29,250 ± 20,260 (*n* = 10; 8638 to 70,691 reads) for the larger space. The maximum number of reads was 70,691, and the minimum was 8638. During alpha diversity and metagenome estimation, 8000 reads per sample were randomly selected and utilized for comparisons among samples. For other analyses, all effective reads were used. These numbers are considered sufficient for analyzing the chicken gut microbiome.

### 3.3. Diversity of Gut Microbiome

**Figure 2:**
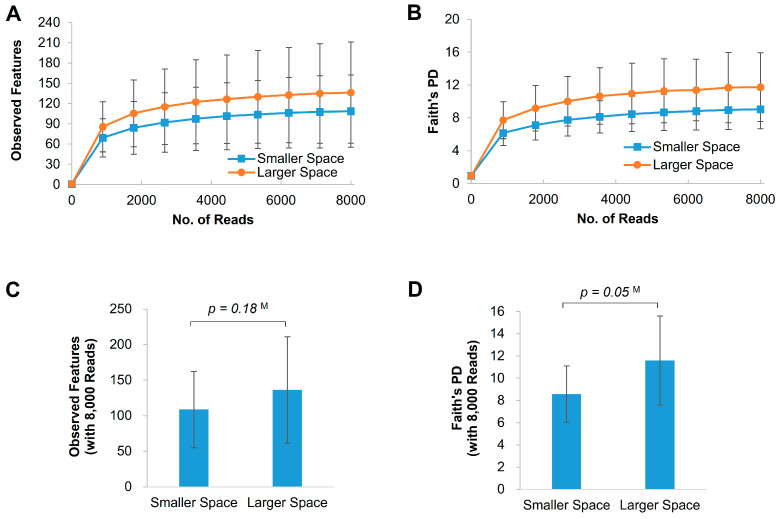
Alpha diversity in fecal microbiota of 39-day-old chickens. Observed features (**A**) and Faith’s PD (**B**) were calculated with different numbers of sequenced reads. Observed features (**C**) and Faith’s PD (**D**) were estimated with 8000 reads for normalization across 10 iterations. The *p*-values (**C**,**D**) were calculated using a one-tailed Mann–Whitney U test (denoted as “M”). Each value is represented as the mean ± standard deviation with error bars.

To evaluate the potential effects of increased housing size on microbial diversity in the gut, we computed alpha diversity indices including observed features and Faith’s PD. Rarefaction curves were plotted for observed features and Faith’s PD with 10 different read numbers (Figure 2A,B). As previously mentioned, we used 8000 reads per sample as the final read point for this analysis. These indices exhibited a gradual increase as the read numbers increased, showing a slight slope after reaching a certain threshold. At the final read number point, the observed features, serving as a measure of richness, did not show a significant increase in the larger space (108.8 ± 53.6 vs. 136.3 ± 75.0, *p* = 0.18, Figure 2C). This suggests that the number of observed microorganism types in the larger space may not be significantly greater than the smaller space. However, Faith’s PD was higher in the larger space, showing marginal significance (8.6 ± 2.5 vs. 11.6 ± 4.0, *p* = 0.05, Figure 2D). This suggests a trend toward greater phylogenetic diversity in the gut microbiome of broilers raised in the larger space.

**Figure 3:**
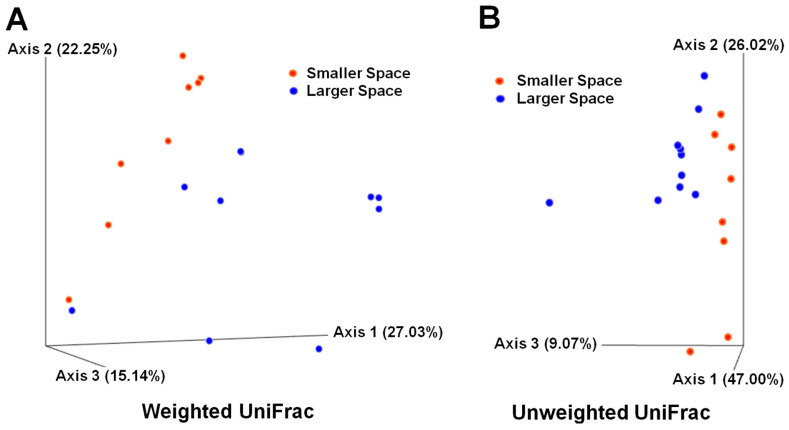
Beta diversity in fecal microbiota of 39-day-old chickens. Weighted (**A**) and unweighted (**B**) UniFrac distances were considered for principal coordinate analysis (PCoA) plots. The percentage of variance explained is shown for each virtual axis. Each filled circle represents an individual chicken sample. All relevant 16S rRNA reads were included in this analysis.

To compare the overall composition of the gut microbiome among samples, we utilized UniFrac distances for beta diversity, either considering microbial abundance (referred to as weighted) or disregarding it (referred to as unweighted). This generated two distinct principal coordinate analysis (PCoA) plots: one based on weighted UniFrac distances (Figure 3A) and another on unweighted UniFrac distances (Figure 3B). A PCoA plot from the unweighted UniFrac data showed that gut microbiome samples from both spaces exhibited significant separation (*p* < 0.05, Figure S1A,C). However, such separation lost statistical significance (*p* = 0.165, Figure S1B,C) when analyzed with the weighted UniFrac data. These results indicate that the distinction between the two groups was primarily driven by the phylogenetic diversity of the gut microbiome, rather than by differences in the abundance of individual microbial features or species.

### 3.4. Relative Abundance of Gut Microbiome

**Figure 4:**
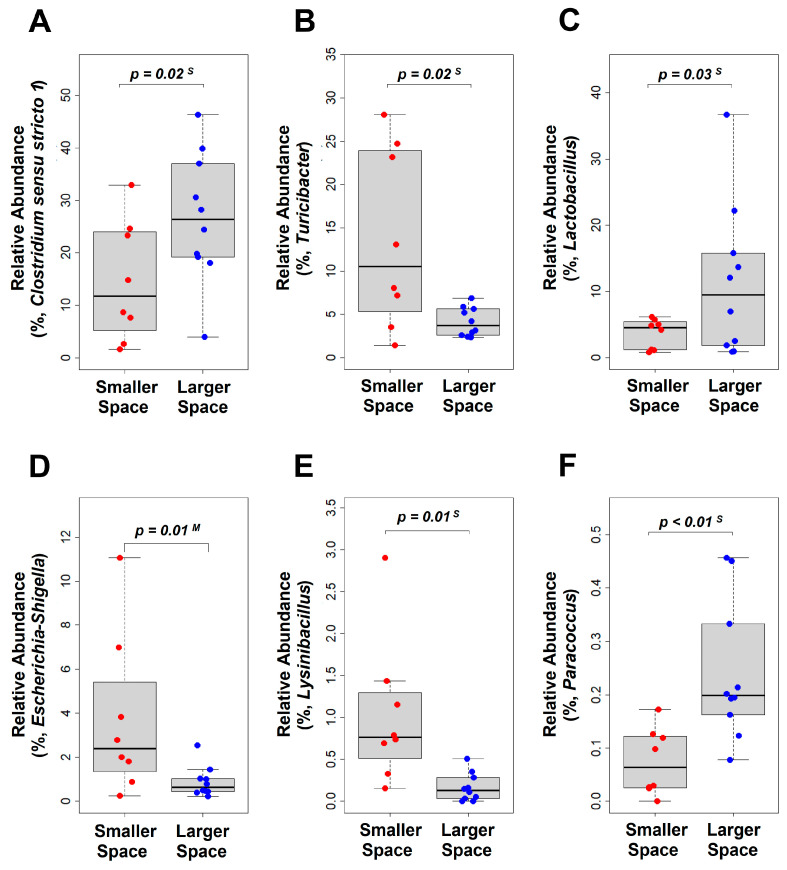
Relative abundance of significant genera. Six genera (**A**–**F**) were selected for representation in the boxplots. Each filled circle represents a value from an individual chicken sample. All relevant reads were included in the analysis. The *p*-values were calculated using either a one-tailed Mann–Whitney U test (denoted as “M”) or a one-tailed Student’s *t*-test (denoted as “S”), following an assessment of normality with the Shapiro–Wilk test.

To identify distinct microbial features that set the two groups apart, we compared the relative abundance of gut microbiome. As shown in Figure S2A, a total of 21 phyla were detected. Among them, one phylum was exclusively associated with the smaller space, thirteen phyla were unique to the larger space, and seven phyla were shared between the two groups. At the phylum level, the combined abundance of Firmicutes, Actinobacteriota, Bacteroidota, and Proteobacteria accounts for a total of 99.45% in both groups (Table 1 and Figure S3A). Notably, Firmicutes constitutes the predominant portion, comprising 83.33% of the total. A total of 258 genera were identified in both groups (Figure S2B). Among them, 20 genera were exclusively present within the confines of the smaller space, while 128 genera were unique to the larger space. Additionally, 110 genera were found to be common to both groups. Notably, the combined relative abundance of 22 major genera encompasses 88.58% in both groups (Table 1 and Figure S3B). Among them, 5 genera such as *Clostridium_sensu_stricto_1*, *Romboutsia*, *Turicibacter*, *Lactobacillus*, and *Enterococcus* exhibit an average abundance exceeding 7.00% across all samples. *Turicibacter* (*p* = 0.02), *Escherichia–Shigella* (*p* = 0.01), and *Lysinibacillus* (*p* = 0.01) exhibited higher abundance in the smaller space, whereas *Clostridium_sensu_stricto_1* (*p* = 0.02), *Lactobacillus* (*p* = 0.03), and *Paracoccus* (*p* < 0.01) showed greater prevalence in the larger space (Table 1 and Figure 4).

The relative abundance of each microbial taxon at the phylum or genus level was subjected to a more detailed analysis to assess its relationship with various factors, including BW, blood cortisol levels, brain *FKBP51* mRNA expression, and other microbial taxa (Figure S4). Numerous pairs of correlations were identified. Notably, the expression level of brain *FKBP51* exhibited a significant positive correlation with the genus *Romboutsia*, with correlation coefficients of 0.921 (*p* < 0.01), 0.662 (*p* = 0.07), and 0.419 (*p* = 0.14) for the smaller space, larger space, and both combined, respectively (Figure S4). Furthermore, six genera, namely *Staphylococcus*, *Corynebacterium*, *Brachybacterium*, *Brevibacterium*, *Dietzia*, and *Lysinibacillus*, exhibit a significant correlation with one another (Figure S3C). For example, notable positive correlations were found between the genera *Lysinibacillus* and *Staphylococcus*, with correlation coefficients of 0.654 (*p* = 0.08 for the smaller space), 0.553 (*p* = 0.10 for the larger space), and 0.733 (*p* < 0.01 for both combined) (Figure S4).

### 3.5. Abundance of Metagenomic Features

**Figure 5:**
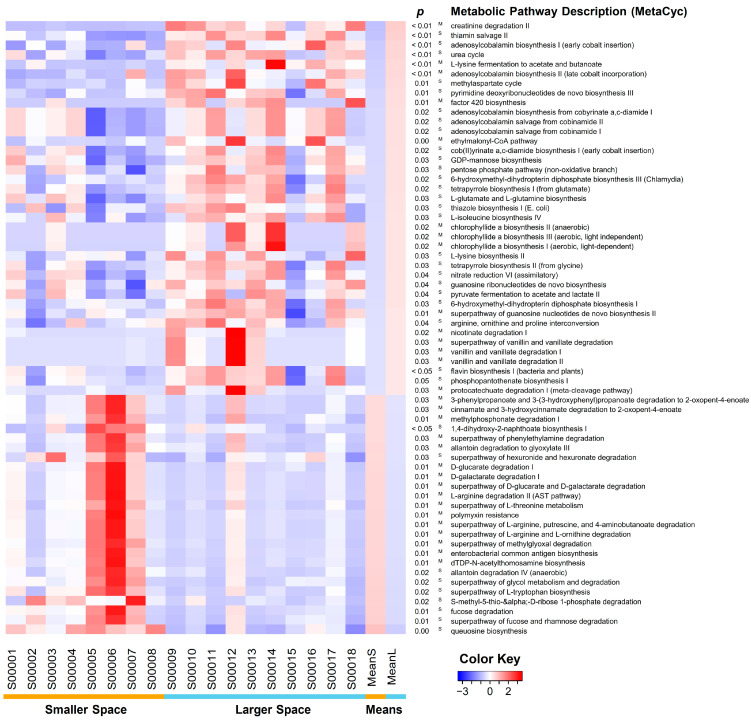
Heatmap of metagenomic abundance. The heatmap summarizes a set of 64 metabolic pathways that exhibit significant differences (*p* < 0.05). The abundance of MetaCyc pathways was estimated using 8000 relevant reads per sample to ensure normalization across samples (S000001–S000018). The terms “MeanS” and “MeanL” correspond to the Z-transformed means of the smaller and larger spaces, respectively. The value of each row was scaled using the Z-transformation, and the rows were sorted based on the MeanS values. The *p*-values were calculated using either a one-tailed Mann–Whitney U test (denoted as “M”) or a one-tailed Student’s *t*-test (denoted as “S”), following an assessment of normality with the Shapiro–Wilk test.

To identify metagenomic changes by the increased housing size, MetaCyc metabolic pathways as metagenomic features were predicted based on 16S rRNA sequences. A total of 389 MetaCyc pathways were identified across the two groups. Among them, the abundance of 64 MetaCyc pathways differed significantly between the two groups (Figure 5 and Table S1). The smaller space was associated with an increased abundance in 25 MetaCyc pathways. Notably, pathways related to queuosine biosynthesis (*p* < 0.01), fucose degradation (*p* = 0.01), allantoin degradation (*p* < 0.05), L-tryptophan biosynthesis (*p* = 0.02), L-threonine degradation (*p* = 0.01), and L-arginine degradation (*p* = 0.01) exhibited significantly higher representation in the smaller space. In contrast, the larger space showed an increased abundance in 39 MetaCyc pathways. Pathways such as creatinine degradation (*p* < 0.01), thiamin (*p* < 0.01), and thiazole (*p* = 0.03) biosynthesis for vitamin B1; adenosylcobalamin (*p* < 0.05) and tetrapyrrole (*p* < 0.05) biosynthesis for vitamin B12 (cobalamin); urea cycle (*p* < 0.01); GDP-mannose biosynthesis (*p* = 0.03); L-lysine fermentation (*p* < 0.01) and biosynthesis (*p* = 0.03); L-isoleucine biosynthesis (*p* = 0.03); and L-glutamate/L-glutamine biosynthesis (*p* = 0.03) were significantly more prevalent in the larger space. Many of the pathways that exhibited significant differences between the two groups are associated with protein metabolism.

## 4. Discussion

Blood cortisol levels are frequently used as an indicator of acute stress in broilers. For instance, its levels decreased in electrically stunned broilers during short slaughter, indicating its association with acute stress [20]. Blood corticosterone levels are also recognized as another biomarker for broiler stress. For example, 42-day-old broilers exposed to HSD (18.8 birds/m^2^) for 14 days as a form of chronic stress showed increased blood corticosterone levels [21]. The mRNA expression level of the *FKBP51* gene in the brain is widely recognized for its association with depression in humans [22,23], corticosteroid responsiveness (as a sensitive biomarker) [24], and acute stress in chickens [25]. In this study, no significant changes were observed in the blood cortisol levels and brain *FKBP51* mRNA expression. These parameters may not be suitable for assessing long-term stress. Further research is needed to better understand the effects of increased housing size on chronic stress, with alternative indicators such as blood corticosterone and feather cortisol [26] potentially offering greater sensitivity.

A previous study on broilers raised at a density of 12.1 birds/m^2^, comparable to ours, reported that at 4–5 weeks of age, the broilers preferred the center of the pen [8]. However, by 6 weeks of age, their preference shifted toward the pen walls. Similarly, in our study, wall preference was also observed toward the end of the rearing period, although it was not explicitly quantified. Despite the same stocking densities between the two groups, broilers that remained near the pen walls in the larger space had better access to a more spacious central area, which may have provided greater comfort for movement and resting. This spatial distribution may have contributed to positive effects on BW and the gut microbiome. Further studies are needed to investigate the relationship between these behavioral patterns and phenotypic outcomes in more detail.

Previous studies [5,6] reported that when 7-day-old broilers were raised under HSD (22 birds/m^2^) for 6 weeks, BW and alpha diversity were reduced compared to normal stocking density (NSD, 14 birds/m^2^), while the abundances of *Turicibacter* and pathogenic *Shigella* increased. These adverse effects of HSD were alleviated by supplementation with a feed additive, chlorogenic acid, which increased BW and alpha diversity and decreased the abundances of these two genera, resulting in patterns similar to those observed under NSD. In our study, comparable results were observed: the smaller space exhibited changes in BW, Faith’s PD (despite the marginal significance), and the two genera, which closely resemble those observed under HSD in previous broiler studies. Furthermore, our housing space strategy led to improvements in these parameters, resembling the effects of feed additive intervention. These findings suggest that such comparable improvements may be achieved through simple environmental modifications without relying on a specific feed additive. To validate this hypothesis, future studies are needed to directly compare the effects of environmental modifications with those of feed additives.

Our results on the alpha diversity of the gut microbiome support that the increased housing size influenced the diversity rather than the richness, even when the stocking density remains the same. An increase in Faith’s PD was associated with the presence of 1 + 13 phyla and 20 + 128 genera that were exclusively found in either of the two groups. Additionally, beta diversity analysis based on UniFrac distance revealed a statistically significant separation between the two groups, although this was only evident in the unweighted PCoA plot. Taken together, these results suggest that the unique microbial taxa, rather than relative abundances, played a key role in the observed separation in the PCoA plot. Further research is needed to clarify the roles of these unique taxa in the broiler gut.

Certain bacterial genera, including *Streptococcus*, *Staphylococcus*, *Escherichia*, and *Shigella*, are widely recognized as pathogenic or harmful to both human and animal health [27,28,29]. Among these, the *Escherichia–Shigella* group showed a significant reduction in the larger space (*p* = 0.01), whereas reductions in the other genera were not statistically significant.

A previous mouse (7–8-week-old C57BL/6) study reported reductions in BW and the relative abundance of *Clostridium sensu stricto 1*, *Romboutsia*, and *Lactobacillus* under the chronic restraint stress (CRS) for two weeks [30]. *Lactobacillus* is a genus of lactic acid bacteria that is considered beneficial in the guts of both humans and animals [10,13]. *Clostridium sensu stricto 1* and *Romboutsia* are well-known as major commensals of the chicken gut [13], as well as butyrate producers, which are recognized for their anti-inflammatory effects in the gut [31]. *Romboutsia* progressively increased in the gut of egg-laying hens over the course of 58 days of growth as a gut commensal microorganism [13]. We observed CRS-like effects, such as comparable decreases in BW and in the two genera (commensal *Clostridium sensu stricto 1* and beneficial *Lactobacillus*) in the smaller space, suggesting that the broilers still experience environmental stress due to closer interactions or space limitations. In contrast, the larger space appeared to provide a more comfortable environment, which was sufficient to increase BW and the abundance of the two genera in broilers.

We observed a higher relative abundance of *Lysinibacillus* in the smaller space, along with its positive correlation with pathogenic *Staphylococcus* when data from both spaces were combined (Figure S4C). Notably, three chickens with the highest relative abundance of *Lysinibacillus* were also lighter than the average BW. In addition to our findings, previous studies, including our own, have reported the following observations. Certain species of *Lysinibacillus* have been identified as potential probiotics for mice [32] and fish [33]. In chickens infected with a low-pathogenic avian influenza virus (H9N2) at 3 weeks of age, the abundance of *Lysinibacillus* in the colon was reported to increase at 2 and 4 days post-infection [34,35]. However, oral administration of probiotic *Enterococcus faecium* (JB00008) cells [36] or a postbiotic preparation of *Lactococcus lactis* (IL1403) [37] has been shown to reduce the abundance of *Lysinibacillus* in the gut of broilers. Furthermore, the abundance of *Lysinibacillus* was reported to be negatively correlated with BW in 58-day-old egg layers [13]. Like many other gut bacterial genera, the role of *Lysinibacillus* strains in the gut remains poorly understood, and it is unclear whether they are beneficial or harmful. Based on our findings and previous observations, we propose that certain *Lysinibacillus* species may have negative effects on broiler health and growth.

We also observed several differences in metagenomic features between the two groups (Figure 5 and Table S1). Queuosine biosynthesis and fucose degradation were higher in the smaller space. Queuosine, which is synthesized from queuine, is well-known for improving translation accuracy [38]. As both queuosine and queuine, which are essential in mammals, are highly dependent on the intestinal microbiome, a lack of tRNA modification by queuine has been known to be linked to the development of inflammatory bowel disease [38]. Fucose utilizers protect the host from infections by pathogens [39,40]. However, the reasons for the higher levels of these features in the smaller space remain unclear. In contrast, in the larger space, biosynthesis pathways for several amino acids and the urea cycle were more prominent. These pathways are involved in protein metabolism. Since the urea cycle is incomplete in birds due to the absence of an essential enzyme, the microbial urea cycle plays a key role in the utilization of excess nitrogen in chickens after microbial intervention in the gut [41]. Based on such observations, we hypothesized that the amino acids and ammonia produced by the gut microbiome may contribute to the higher BW or growth observed in the larger group. However, individual features related to amino acid biosynthesis were not positively correlated with BW.

## 5. Conclusions

In this study, even with the same stocking density, broilers raised in the smaller space still exhibited biological issues related to growth and the gut microbiome. Our strategy, which involved a simple intervention of increasing housing space without altering stocking density to modify the rearing environment, demonstrated the potential to partially alleviate these issues by improving body weight and the gut microbiome. Future studies should investigate the application of this strategy at higher stocking densities to improve both the environmental conditions and productivity of chickens.

## Figures and Tables

**Table 1:** Relative abundance of major phyla and genera ^1^.

Taxa ^2^	Smaller Space (%) ^3^	Larger Space (%) ^4^	*p*-Values ^5^
Phyla			
Firmicutes	81.31 ± 14.01	84.95 ± 10.65	0.28 ^S^
Actinobacteriota	7.16 ± 7.87	6.45 ± 5.41	0.42 ^S^
Bacteroidota	6.83 ± 11.82	4.54 ± 6.98	0.29 ^M^
Proteobacteria	4.46 ± 4.08	3.26 ± 5.19	0.12 ^M^
Genera			
*Clostridium_sensu_stricto_1*	14.55 ± 11.39	26.76 ± 12.40	0.02 ^S^
*Romboutsia*	10.89 ± 5.86	14.55 ± 6.35	0.11 ^S^
*Turicibacter*	13.65 ± 10.33	4.12 ± 1.66	0.02 ^S^
*Lactobacillus*	3.64 ± 2.21	11.36 ± 11.52	0.03 ^S^
*Enterococcus*	11.47 ± 11.71	4.53 ± 3.36	0.26 ^M^
*Bacteroides*	6.83 ± 11.82	3.44 ± 6.48	0.18 ^M^
*Cellulosilyticum*	4.15 ± 3.62	4.21 ± 2.74	0.49 ^S^
*Terrisporobacter*	3.36 ± 2.47	4.75 ± 2.67	0.03 ^M^
*Corynebacterium*	3.60 ± 4.10	3.21 ± 2.70	0.52 ^M^
*Streptococcus*	4.97 ± 6.82	2.00 ± 0.93	0.41 ^M^
*Staphylococcus*	4.84 ± 5.57	1.27 ± 1.11	0.32 ^M^
*Escherichia–Shigella*	3.70 ± 3.63	0.88 ± 0.69	0.01 ^M^
*Clostridia_vadinBB60_group*	1.15 ± 2.67	2.16 ± 5.12	0.12 ^M^
*Brachybacterium*	1.60 ± 1.62	1.40 ± 1.22	0.48 ^M^
*Brevibacterium*	1.22 ± 1.38	0.87 ± 0.79	0.42 ^M^
*Lysinibacillus*	1.02 ± 0.86	0.16 ± 0.17	0.01 ^S^
*Ruminococcus*	0.21 ± 0.41	0.24 ± 0.43	0.66 ^M^
*Paracoccus*	0.07 ± 0.06	0.24 ± 0.13	<0.01 ^S^
*Bacillus*	0.27 ± 0.62	0.08 ± 0.10	0.44 ^M^
*Faecalibacterium*	0.25 ± 0.59	0.07 ± 0.12	0.39 ^M^
*Oscillospira*	0.00 ± 0.01	0.01 ± 0.02	0.56 ^M^
*Lactococcus*	0.00 ± 0.01	0.00 ± 0.00	0.18 ^M^

^1^ These data were from all relevant reads. ^2^ We focused on microbial taxa with a relative abundance greater than 1% or those of significant biological importance. ^3,4^ Data are presented as mean ± SD. ^5^ The *p*-values were calculated using either a one-tailed Mann–Whitney U test (denoted as “M”) or a one-tailed Student’s *t*-test (denoted as “S”), following an assessment of normality with the Shapiro–Wilk test.

## Data Availability

The sequenced reads in this study are deposited in the NCBI’s Sequence Read Archive (SRA) under the accession number PRJNA1207695.

## Supplementary Materials

The following supporting information can be downloaded at: https://www.mdpi.com/article/10.3390/ani15030441/s1. Figure S1: Statistics of UniFrac distances. Figure S2: Detected and shared taxa. Figure S3: Relative abundance of major bacterial groups. Figure S4: Correlation matrices for relative abundance and other parameters. Table S1: Metagenomic abundance of gut microbiome.
