# Supplementary material for: Effects of Increased Housing Space Without Altering Stocking Density on Body Weight, Stress, and Gut Microbiome in Broiler Chickens
Source: Animals (Basel). 2025 Feb 5;15(3):441. doi: 10.3390/ani15030441 (PMC11816066; doi:10.3390/ani15030441)
Supplement: Supplementary file 1 [file animals-15-00441-s001.zip › animals-3444433-supplementary.pdf]

Supplementary Materials

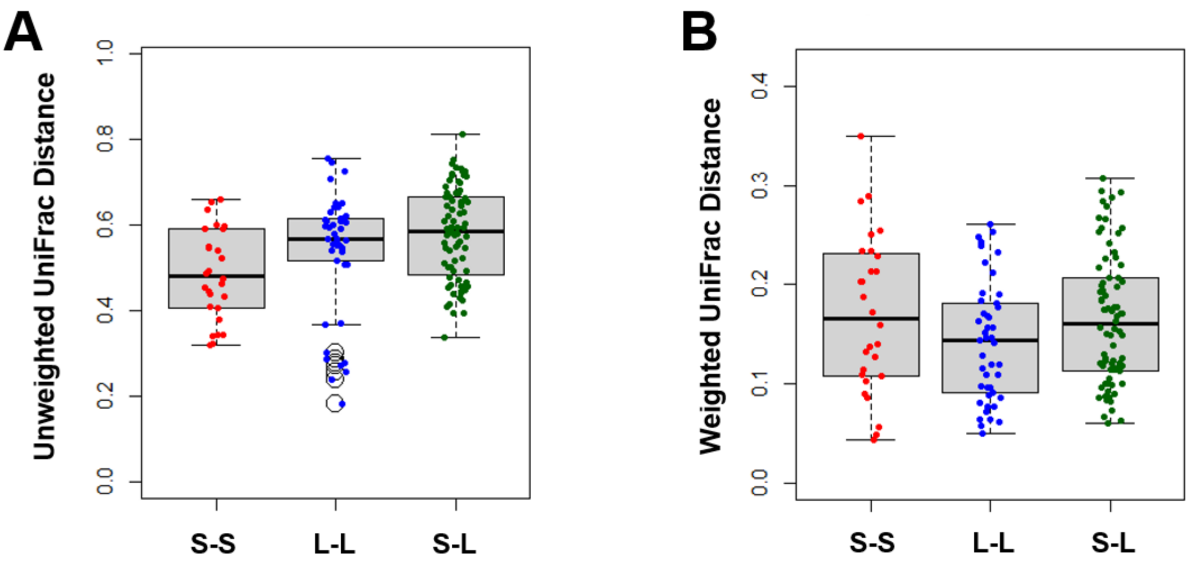

C

UniFrac	Group 1	Group 2	Sample size	Permutations	Pseudo-F	P-value	Q-value
Unweighted	Smaller Space	Larger Space	18	999	3.067	0.003	0.003
Weighted	Smaller Space	Larger Space	18	999	1.688	0.165	0.165

Figure S1. Statistics of UniFrac distances. Unweighted (A) and weighted (B) UniFrac distances were visualized using boxplots, with each dot representing the UniFrac distance between a pair of different samples. S-S (*n* = 28), L-L (*n* = 45), and S-L (*n* = 80) correspond to sample pairs from the smaller (S) or larger (L) spaces. Pairwise PERMANOVA statistics were calculated using 999 permutations (C).

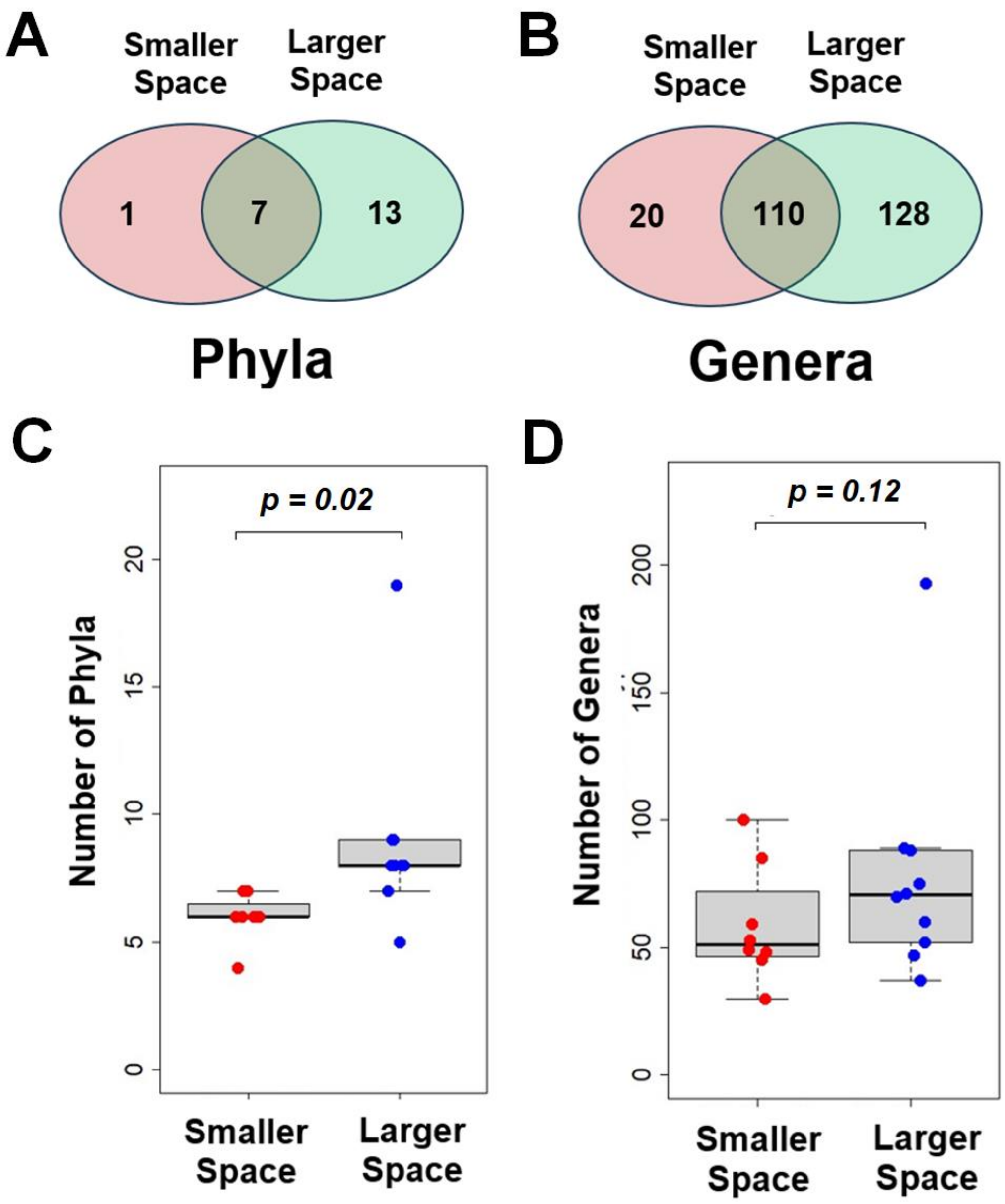

Figure S2. Detected and shared taxa. Twenty-one phyla (A) and 258 genera (B) were identified across the two groups and presented using Venn diagrams. The numbers in the distinct sections of the diagrams represent the count of phyla or genera. The phyla (C) and genera (D) found in each group were compared. Each dot represents an individual sample. All relevant reads were included in the analyses. *p*-values (C and D) were calculated using a one-tailed Student's t-test.

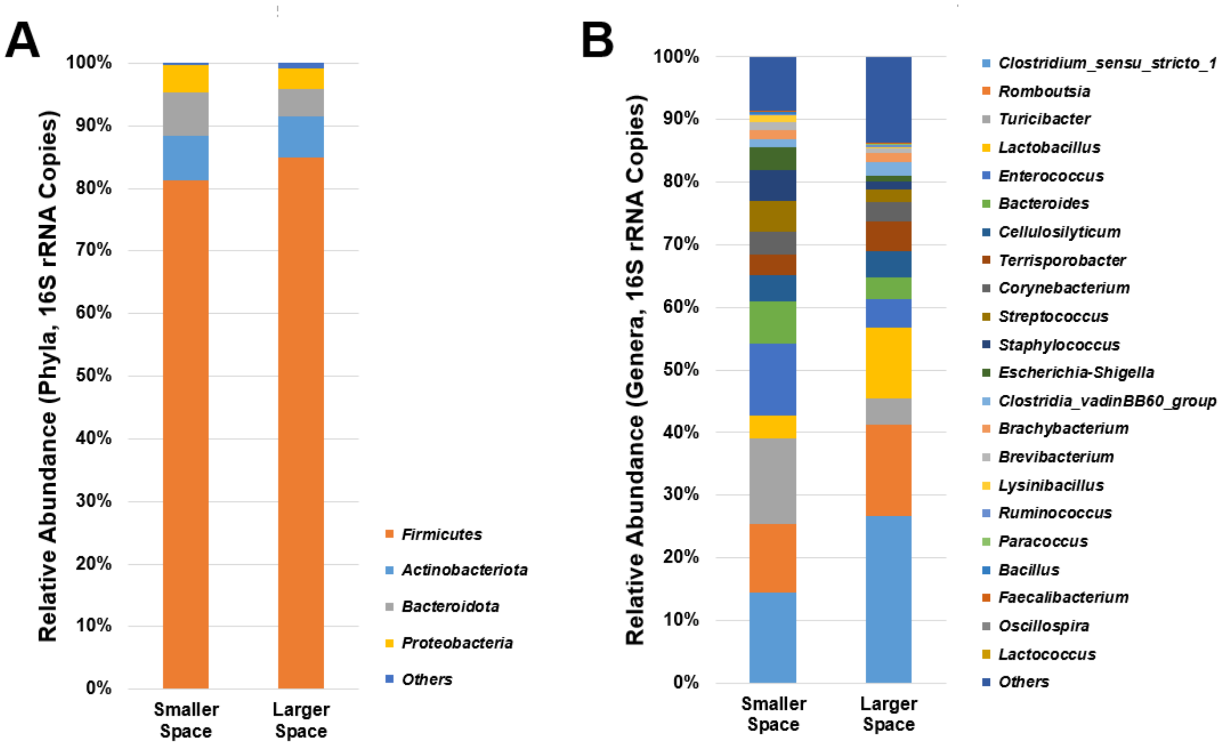

Figure S3. Relative abundance of major bacterial groups. Relative abundance was determined by the copy number of 16S rRNA reads and presented at the phylum (A) and genus (B) levels. The abundance values represent the average for each microbial group.

A

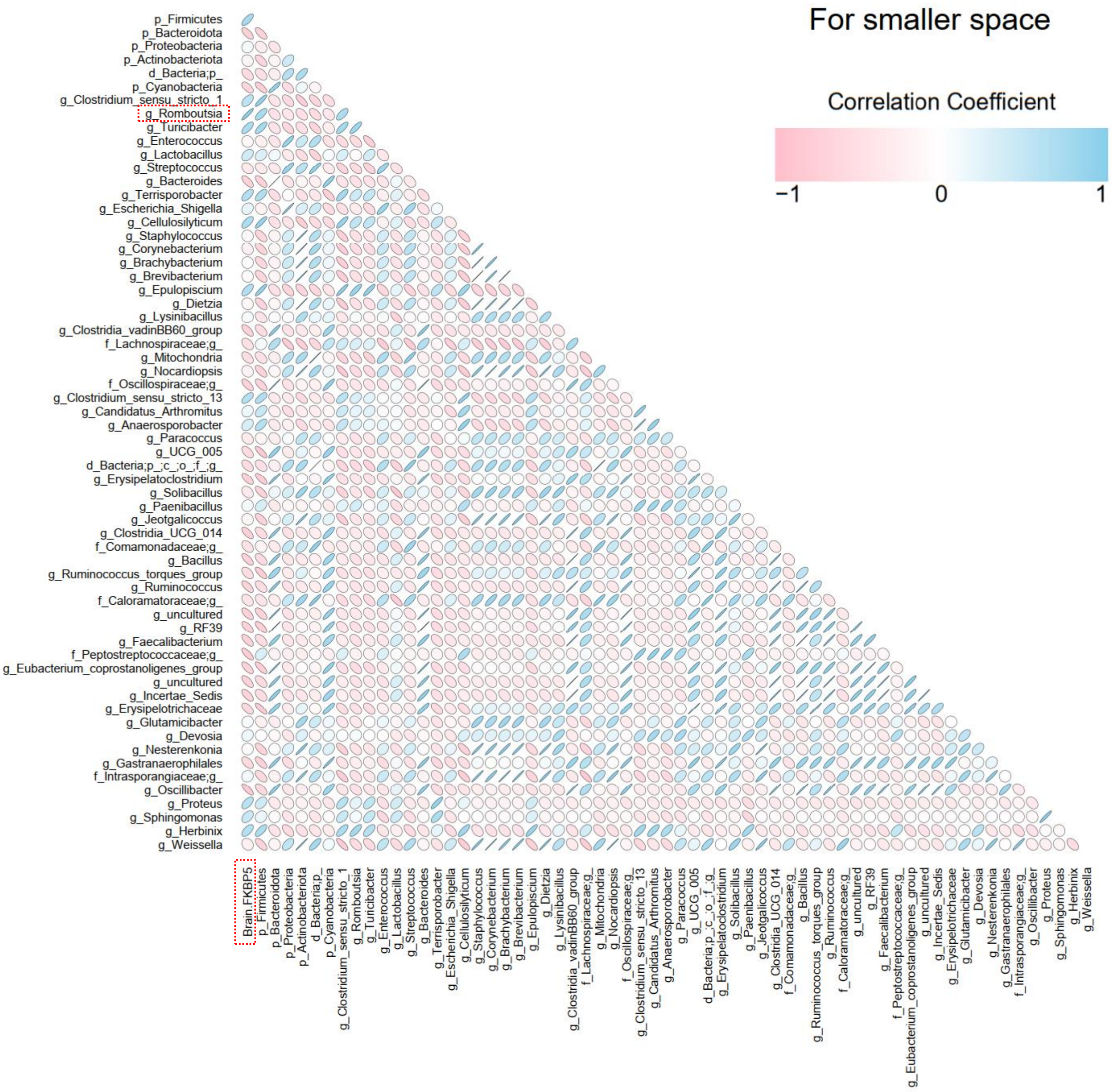
For smaller space
Correlation Coefficient
−1
0
1
Brain.FKBP5
g_Romboutsia

B

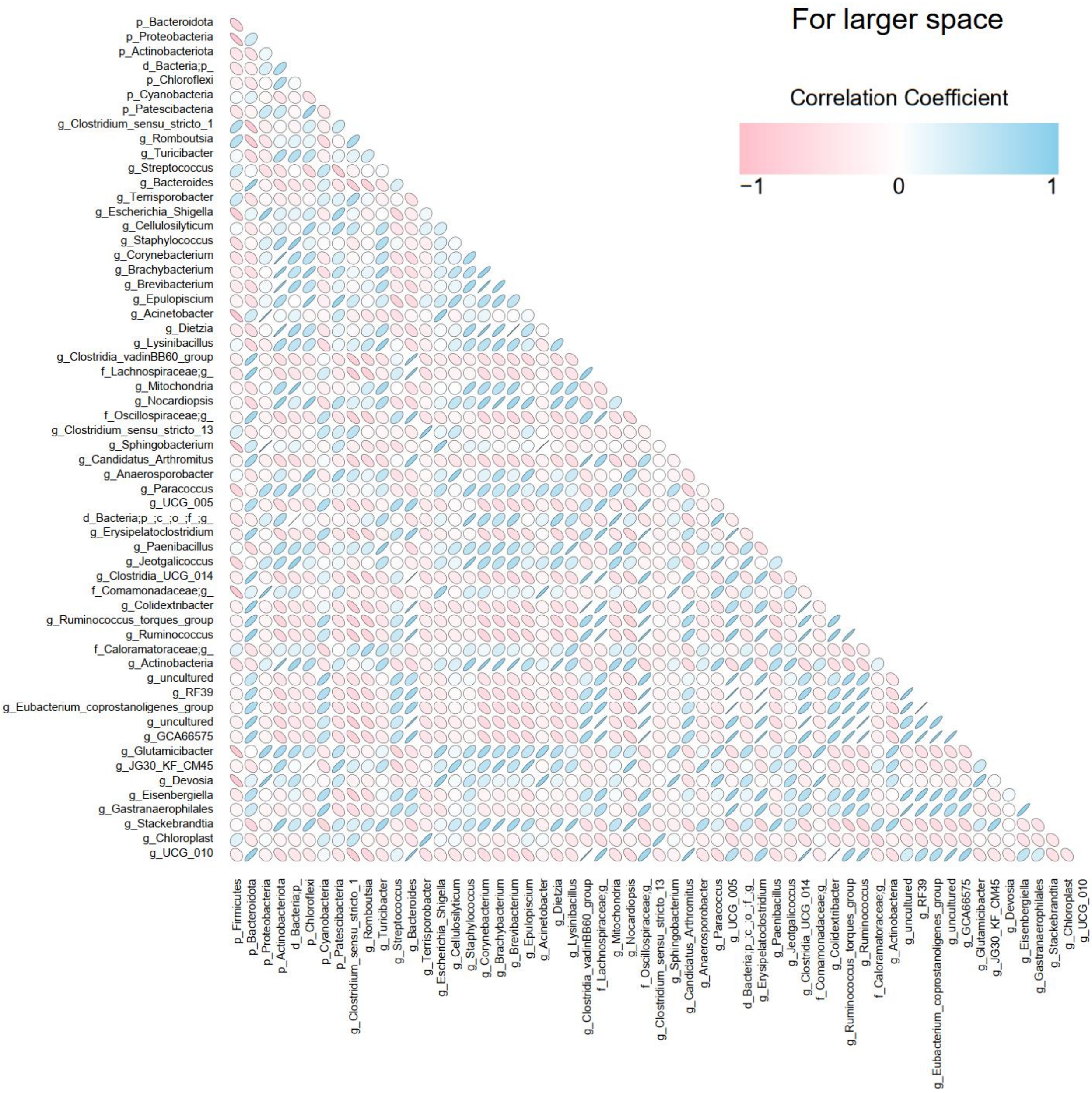
For larger space
Correlation Coefficient
−1
0
1
p_Bacteroidota
p_Proteobacteria
p_Actinobacteriota
d_Bacteria;p_
p_Chloroflexi
p_Cyanobacteria
p_Patescibacteria
g_Clostridium_sensu_stricto_1
g_Romboutsia
g_Turicibacter
g_Streptococcus
g_Bacteroides
g_Terrisporobacter
g_Escherichia_Shigella
g_Cellulosilyticum
g_Staphylococcus
g_Corynebacterium
g_Brachybacterium
g_Brevibacterium
g_Epulopiscium
g_Acinetobacter
g_Dietzia
g_Lysinibacillus
g_Clostridia_vadinBB60_group
f_Lachnospiraceae;g_
g_Mitochondria
g_Nocardiopsis
f_Oscillospiraceae;g_
g_Clostridium_sensu_stricto_13
g_Sphingobacterium
g_Candidatus_Arthromitus
g_Anaerosporobacter
g_Paracoccus
g_UCG_005
d_Bacteria;p_;c_;o_;f_;g_
g_Erysipelatoclostridium
g_Paenibacillus
g_Jeotgalicoccus
g_Clostridia_UCG_014
f_Comamonadaceae;g_
g_Colidextribacter
g_Ruminococcus_torques_group
g_Ruminococcus
f_Caloramatoraceae;g_
g_Actinobacteria
g_uncultured
g_RF39
g_Eubacterium_coprostanoligenes_group
g_uncultured
g_GCA66575
g_Glutamicibacter
g_JG30_KF_CM45
g_Devosia
g_Eisenbergiella
g_Gastranaerophilales
g_Stackebrandtia
g_Chloroplast
g_UCG_010
p_Firmicutes
p_Bacteroidota
p_Proteobacteria
p_Actinobacteriota
d_Bacteria;p_
p_Chloroflexi
p_Cyanobacteria
p_Patescibacteria
g_Clostridium_sensu_stricto_1
g_Romboutsia
g_Turicibacter
g_Streptococcus
g_Bacteroides
g_Terrisporobacter
g_Escherichia_Shigella
g_Cellulosilyticum
g_Staphylococcus
g_Corynebacterium
g_Brachybacterium
g_Brevibacterium
g_Epulopiscium
g_Acinetobacter
g_Dietzia
g_Lysinibacillus
g_Clostridia_vadinBB60_group
f_Lachnospiraceae;g_
g_Mitochondria
g_Nocardiopsis
f_Oscillospiraceae;g_
g_Clostridium_sensu_stricto_13
g_Sphingobacterium
g_Candidatus_Arthromitus
g_Anaerosporobacter
g_Paracoccus
g_UCG_005
d_Bacteria;p_;c_;o_;f_;g_
g_Erysipelatoclostridium
g_Paenibacillus
g_Jeotgalicoccus
g_Clostridia_UCG_014
f_Comamonadaceae;g_
g_Colidextribacter
g_Ruminococcus_torques_group
g_Ruminococcus
f_Caloramatoraceae;g_
g_Actinobacteria
g_uncultured
g_RF39
g_Eubacterium_coprostanoligenes_group
g_uncultured
g_GCA66575
g_Glutamicibacter
g_JG30_KF_CM45
g_Devosia
g_Eisenbergiella
g_Gastranaerophilales
g_Stackebrandtia
g_Chloroplast
g_UCG_010

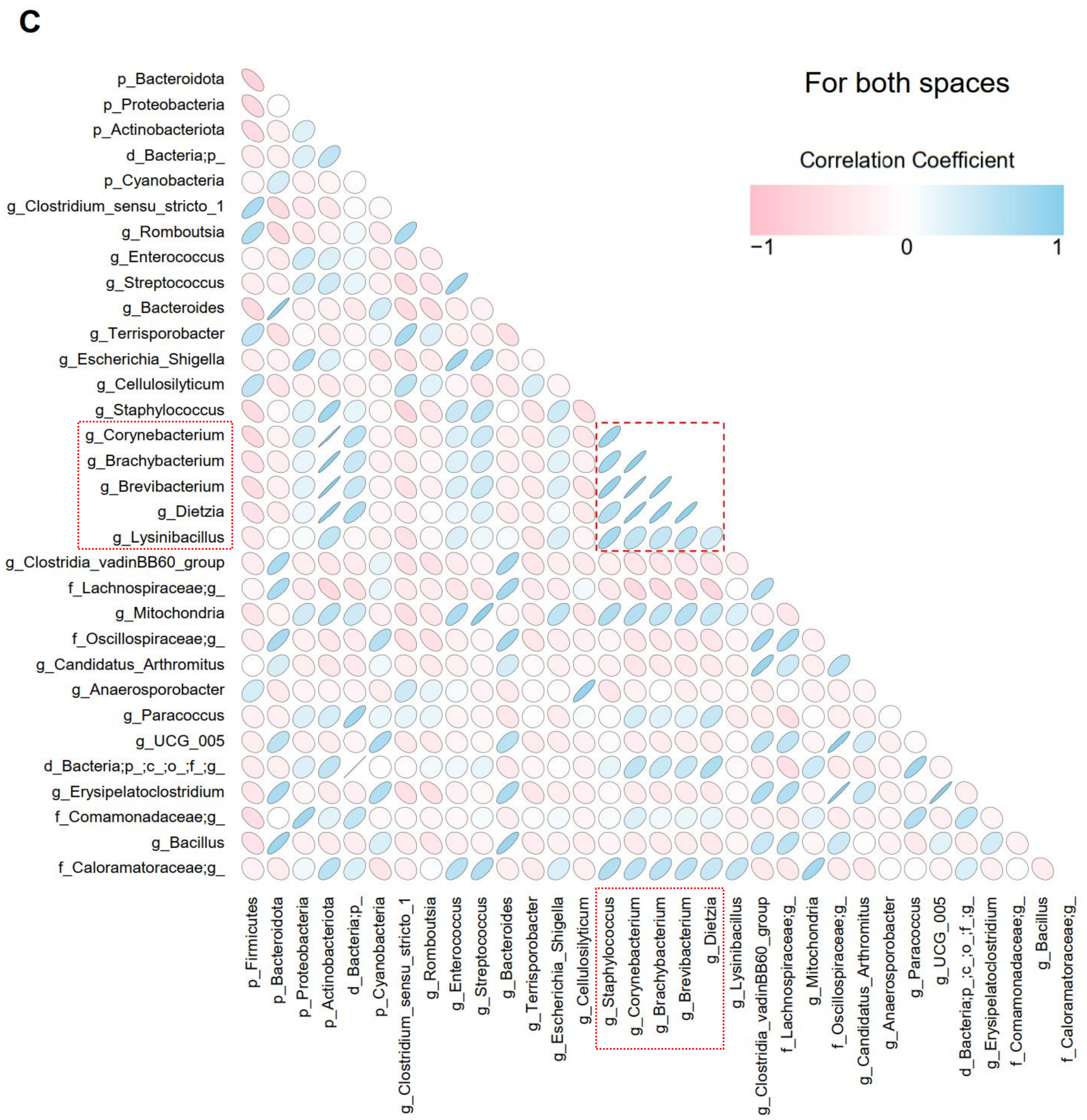

Figure S4. Correlation matrices for relative abundance and other parameters. The parameters on the left and bottom of the correlation matrix were selected based on specific criteria: correlation coefficient ($-0.7 < r < 0.7$), *p*-value (< 0.1), and, when applicable, relative abundance (> 0.01%). The color and intensity of the ovals represent the correlation strength for each parameter pair, as indicated by the intensity bar. Symbols such as p_, c_, o_, f_, and g_ denote different taxonomic ranks: phylum, class, order, family, and genus, respectively. Symbols without accompanying names indicate unclassified taxa. The data analyzed were derived from all relevant reads. Correlation matrices are displayed for the smaller space (A), larger space (B), and both spaces combined (C).

Table S1. Metagenomic abundance of gut microbiome.

No.	MetaCyc Pathways	Gene Abundance		p-values[1]		Comparison
		Smaller Space (SS)	Larger Space (LS)			
1	creatinine degradation II	2.5 ± 3.2	86.3 ± 46.2	< 0.01	M	SS < LS
2	thiamin salvage II	4360.2 ± 551	5632.3 ± 607.1	< 0.01	S	SS < LS
3	adenosylcobalamin biosynthesis I (early cobalt insertion)	217.2 ± 215	620.8 ± 263.8	< 0.01	S	SS < LS
4	urea cycle	3313.5 ± 837.7	4667.5 ± 827.1	< 0.01	S	SS < LS
5	L-lysine fermentation to acetate and butanoate	26.7 ± 56.1	220.8 ± 175.7	< 0.01	M	SS < LS
6	adenosylcobalamin biosynthesis II (late cobalt incorporation)	132 ± 171.9	392.7 ± 206	< 0.01	M	SS < LS
7	methylaspartate cycle	46 ± 35.9	186.9 ± 138.1	0.01	S	SS < LS
8	pyrimidine deoxyribonucleotides de novo biosynthesis III	2878.8 ± 263.9	3515.1 ± 687.4	0.01	S	SS < LS
9	factor 420 biosynthesis	0.9 ± 2.4	7.1 ± 6.9	0.01	M	SS < LS
10	adenosylcobalamin biosynthesis from cobyrinate a,c-diamide I	3473.9 ± 1278.9	4687.3 ± 966.6	0.02	S	SS < LS
11	adenosylcobalamin salvage from cobinamide II	3491.9 ± 1277.7	4695.8 ± 966.8	0.02	S	SS < LS
12	adenosylcobalamin salvage from cobinamide I	3576.7 ± 1236.2	4741.4 ± 961.7	0.02	S	SS < LS
13	ethylmalonyl-CoA pathway	18 ± 14.8	67.7 ± 61.4	0.00	M	SS < LS
14	cob(II)yrinate a,c-diamide biosynthesis I (early cobalt insertion)	2669.1 ± 1341.6	3989.4 ± 1183.7	0.02	S	SS < LS
15	GDP-mannose biosynthesis	4749.4 ± 906.5	5562.9 ± 675.1	0.03	S	SS < LS
16	pentose phosphate pathway (non-oxidative branch)	9639.5 ± 1174.2	10706.1 ± 929.7	0.03	S	SS < LS
17	6-hydroxymethyl-dihydropterin diphosphate biosynthesis III (Chlamydia)	4168.8 ± 346.7	4965.1 ± 994.4	0.02	S	SS < LS
18	tetrapyrrole biosynthesis I (from glutamate)	3779.2 ± 795.8	4836.9 ± 1191.2	0.02	S	SS < LS
19	L-glutamate and L-glutamine biosynthesis	3439.8 ± 1935.9	5050.2 ± 1301.7	0.03	S	SS < LS
20	thiazole biosynthesis I (E. coli)	2830 ± 563	3367.6 ± 529.5	0.03	S	SS < LS
21	L-isoleucine biosynthesis IV	6878.2 ± 1129	8028.4 ± 1230.7	0.03	S	SS < LS
22	chlorophyllide a biosynthesis II (anaerobic)	0.8 ± 2.2	8 ± 9.9	0.02	M	SS < LS
23	chlorophyllide a biosynthesis III (aerobic, light independent)	0.8 ± 2.2	8 ± 9.9	0.02	M	SS < LS
24	chlorophyllide a biosynthesis I (aerobic, light-dependent)	0.8 ± 2.2	7.2 ± 8.8	0.02	M	SS < LS
25	L-lysine biosynthesis II	3864.4 ± 598	4729.2 ± 1099.8	0.03	S	SS < LS
26	tetrapyrrole biosynthesis II (from glycine)	3718 ± 859.1	4748.1 ± 1259.4	0.03	S	SS < LS
27	nitrate reduction VI (assimilatory)	3108.4 ± 1827.5	4735.1 ± 1684.9	0.04	S	SS < LS
28	guanosine ribonucleotides de novo biosynthesis	7089.9 ± 552	7536.6 ± 436.5	0.04	S	SS < LS
29	pyruvate fermentation to acetate and lactate II	8765.3 ± 1925.6	10364.5 ± 1619.1	0.04	S	SS < LS
30	6-hydroxymethyl-dihydropterin diphosphate biosynthesis I	4154.2 ± 390.2	4862.7 ± 1002.6	0.03	S	SS < LS
31	superpathway of guanosine nucleotides de novo biosynthesis II	5315.4 ± 497.1	5991.2 ± 974.4	0.01	M	SS < LS
32	arginine, ornithine and proline interconversion	1927.4 ± 519.7	2437 ± 670.3	0.04	S	SS < LS
33	nicotinate degradation I	1.2 ± 2.3	17.2 ± 26	0.02	M	SS < LS
34	superpathway of vanillin and vanillate degradation	0.3 ± 0.8	13.5 ± 21.6	0.03	M	SS < LS
35	vanillin and vanillate degradation I	0.3 ± 0.8	13.5 ± 21.6	0.03	M	SS < LS
36	vanillin and vanillate degradation II	0.3 ± 0.8	14.9 ± 23.9	0.03	M	SS < LS
37	flavin biosynthesis I (bacteria and plants)	5100 ± 477.9	5719.9 ± 937.7	< 0.05	S	SS < LS
38	phosphopantothenate biosynthesis I	4604.9 ± 517.7	5308.2 ± 1075.6	0.05	S	SS < LS
39	protocatechuate degradation I (meta-cleavage pathway)	3 ± 4.5	23.9 ± 34.6	0.03	M	SS < LS
40	3-phenylpropanoate and 3-(3-hydroxyphenyl)propanoate degradation to 2-oxopent-4-er	350.4 ± 327	121.2 ± 127.5	0.03	M	SS > LS
41	cinnamate and 3-hydroxycinnamate degradation to 2-oxopent-4-enoate	350.4 ± 327	121.2 ± 127.5	0.03	M	SS > LS
42	methylphosphonate degradation I	462.2 ± 317.4	222.5 ± 173.3	0.01	M	SS > LS
43	1,4-dihydroxy-2-naphthoate biosynthesis I	1711.1 ± 1380.3	773.3 ± 376.3	< 0.05	S	SS > LS
44	superpathway of phenylethylamine degradation	405.7 ± 349.4	151.1 ± 151.5	0.03	M	SS > LS
45	allantoin degradation to glyoxylate III	422.2 ± 330.5	178.4 ± 136	0.03	M	SS > LS
46	superpathway of hexuronide and hexuronate degradation	1461 ± 610.5	959.9 ± 347.4	0.03	S	SS > LS
47	D-glucarate degradation I	387.5 ± 346.3	125.9 ± 95.1	0.01	M	SS > LS
48	D-galactarate degradation I	363 ± 341.3	101.5 ± 84	0.01	M	SS > LS
49	superpathway of D-glucarate and D-galactarate degradation	363 ± 341.3	101.5 ± 84	0.01	M	SS > LS
50	L-arginine degradation II (AST pathway)	296 ± 292.3	73 ± 63.7	0.01	M	SS > LS
51	superpathway of L-threonine metabolism	657.9 ± 500.6	253.2 ± 187.4	0.01	M	SS > LS
52	polymyxin resistance	300.1 ± 291.8	73.3 ± 54.8	0.01	M	SS > LS
53	superpathway of L-arginine, putrescine, and 4-aminobutanoate degradation	571.1 ± 533.4	151.9 ± 119.7	0.01	M	SS > LS
54	superpathway of L-arginine and L-ornithine degradation	571.1 ± 533.4	151.9 ± 119.7	0.01	M	SS > LS
55	superpathway of methylglyoxal degradation	455 ± 404.3	130.8 ± 88.4	0.01	M	SS > LS
56	enterobacterial common antigen biosynthesis	483.3 ± 439	130.1 ± 94.3	0.01	M	SS > LS
57	dTDP-N-acetylthomosamine biosynthesis	581.9 ± 503.8	168.2 ± 119.9	0.01	M	SS > LS
58	allantoin degradation IV (anaerobic)	456.1 ± 361.2	149.1 ± 96.5	0.02	S	SS > LS
59	superpathway of glycol metabolism and degradation	461 ± 319.9	173 ± 94.2	0.02	S	SS > LS
60	superpathway of L-tryptophan biosynthesis	1974.9 ± 1382.6	693.8 ± 473.8	0.02	S	SS > LS
61	S-methyl-5-thio-α-D-ribose 1-phosphate degradation	98.1 ± 69.7	34.7 ± 19.1	0.02	S	SS > LS
62	fucose degradation	424.6 ± 292.5	139.5 ± 95.7	0.01	S	SS > LS
63	superpathway of fucose and rhamnose degradation	558.9 ± 347.3	211.6 ± 124.6	0.01	S	SS > LS
64	queuosine biosynthesis	3267.5 ± 634.4	1703.4 ± 659.7	0.00	S	SS > LS

[1] The *p*-values were calculated using either a one-tailed Mann–Whitney U test (denoted as “M”) or a one-tailed Student's t-test (denoted as “S”), following an assessment of normality with the Shapiro–Wilk test.
